# paraORA: GPU-Accelerated Exact Over-Representation Analysis for Repeated Queries of Large Gene-Set Libraries

**DOI:** 10.3390/biology15181641

**Published:** 2026-09-17

**Authors:** Zheng Wu, Zejun Zhang, Wenqing Feng, Jinlei Sun, Zhichun Liu, Guoqiang Wang, Yunqing Liu

**Affiliations:** 1 School of Computer Science, Luoyang Institute of Science and Technology, Luoyang 471000, Chinajlsun@lit.edu.cn (J.S.); wgq@lit.edu.cn (G.W.); 2 School of Electrical and Energy Engineering, Shanghai Dianji University, Shanghai 201306, China; 18734968335@163.com

**Keywords:** over-representation analysis, hypergeometric test, gene-set analysis, GPU computing, CUDA, functional enrichment

## Abstract

Over-representation analysis (ORA) is widely used to interpret selected-gene lists, including differentially expressed genes and cell-type marker genes. Unlike ranked-list gene-set enrichment analysis (GSEA), ORA tests whether selected genes are over-represented in predefined gene sets under an explicit background. Repeated analysis of large gene-set libraries, however, can be computationally costly. GPU-accelerated methods such as rapidGSEA mainly target ranked-list GSEA and do not address repeated exact-ORA processing. We developed paraORA to accelerate repeated ORA by preparing a gene-set library once and reusing its compressed representation across queries. In the GPU-exact path, overlap counting, exact hypergeometric testing, Benjamini–Hochberg correction, and odds-ratio calculation are performed on the GPU. Across 425 numerical validation cases on three GPU models, paraORA agreed with CPU-reference results within the prespecified tolerance while preserving statistical decisions and rankings. On an RTX 4090, the CPU/GPU runtime ratio for the complete in-memory workflow increased from 1.89 for one query to 60.10 for 100 queries against 141,002 gene sets, including one-time preparation but excluding file writing. In a separate complete file-to-file benchmark, GPU-exact was 2.55-fold faster than CPU execution. An exploratory limma reanalysis showed direction-specific Hallmark enrichment. paraORA is available as a web service and an open-source package.

## Simple Summary

Researchers often compare selected genes with known groups of genes to understand the biological processes involved in an experiment. Repeating these comparisons across large collections can take considerable time. We developed paraORA to make this work faster by preparing a gene-group collection once, reusing it for subsequent analyses, and performing calculations on graphics-processing hardware. In the numerical tests, the tool preserved the statistical decisions and rankings obtained with the reference method. In a benchmark involving 141,002 gene groups, processing 100 gene lists took about nine seconds, compared with nearly nine minutes using the central processor. These times included preparing the collection but excluded saving results to files. A separate test that included reading inputs and saving results showed a smaller, approximately 2.55-fold speedup. An analysis of public gene-expression data illustrated how the tool can identify biological processes associated with selected genes. Available through a web service and as free software for local use, paraORA can help researchers interpret many gene lists more efficiently when they share the same reference collection.

## 1. Introduction

Functional enrichment analysis connects selected-gene lists to biological pathways, molecular functions, or cellular phenotypes. For a thresholded gene list, over-representation analysis (ORA) tests whether a gene set contains more selected genes than expected under a defined background [1,2]. When the tested gene universe is defined explicitly, the same framework can be applied to public annotations or custom gene-set libraries. Gene set enrichment analysis (GSEA) addresses a different type of input by locating a gene set within an ordered genome-wide statistic [3,4]. paraORA focuses on the former task: one-sided exact ORA for thresholded gene lists.

Each ORA query requires overlap counting, hypergeometric-tail evaluation, and multiple-testing correction across all retained gene sets. When multiple contrasts use the same background and gene-set library, the membership index can be prepared once and reused. The statistical tests must still be performed separately for each query; paraORA accelerates these calculations while avoiding repeated reconstruction of the library.

Several established resources provide gene sets and annotation services. Gene Ontology provides a shared functional vocabulary, while topGO accounts for relationships among terms [5,6]. Enrichr provides interactive gene-list enrichment and a gene-set knowledge-discovery resource [7,8], MSigDB curates widely used gene-set collections [9], g:Profiler supports enrichment and identifier mapping [10], and PANTHER provides functional classification and annotation interfaces [11]. Repeated-query computation is a separate concern when these resources are used across many contrasts.

Software packages provide complementary interfaces for applying these resources. clusterProfiler supports enrichment analysis and multi-list comparison in R [12,13], GOATOOLS provides a Python implementation for Gene Ontology analysis [14], and GSEApy supports gene-set analysis in Python [15]. These tools make ORA straightforward to incorporate into routine bioinformatics workflows. paraORA complements them by retaining the same gene sets and enrichment statistics while reducing repeated library-processing and numerical costs in shared-library exact ORA.

Acceleration strategies depend on the enrichment task. rapidGSEA uses multicore CPUs and CUDA-enabled GPUs for ranking, enrichment scoring, and permutation-based GSEA [16], while fgsea accelerates ranked-list enrichment [17]. GPU ORA is also available in OmicVerse v2.2.0 through a Torch implementation [18]. paraORA instead targets repeated selected-list queries under a fixed explicit background and gene-set library, combining a reusable membership index with device-resident exact hypergeometric testing and BH correction.

paraORA prepares a GMT library under an explicit background, reuses its member index across queries, and provides CPU, GPU-hybrid, and GPU-exact execution paths. We evaluated numerical agreement, a biological case study, repeated-query performance, and complete-workflow costs. A shared-resource rapidGSEA experiment is retained in the supplement to distinguish the ranked-list and selected-list workflows. The within-workflow comparisons are shown in Figure S1. The rapidGSEA C2 single-precision configuration was excluded from the timing comparison because its maximum enrichment-score discrepancy (approximately 4.8 × 10^−5^) exceeded the specified single-precision tolerance (10^−5^).

## 2. Materials and Methods

### 2.1. Inputs and Background Definition

A paraORA analysis takes three explicit inputs: a GMT gene-set library, an analyst-specified background gene list, and one or more selected-gene lists. Identifiers are trimmed and deduplicated. Each gene set is restricted to the supplied background before size filtering, and selected genes outside the background are recorded but excluded from the effective count. Explicit definition of the reference gene universe is important because background specification is a recognized source of inconsistency in functional enrichment analysis [19,20]. Selection-related bias can also affect Gene Ontology analyses of RNA-seq gene lists [21], and enrichment results depend on transparent reporting of analytical choices, including the tested gene universe and selection threshold [22]. All paraORA statistics are therefore calculated within the gene universe specified by the analyst.

**Figure 1:**
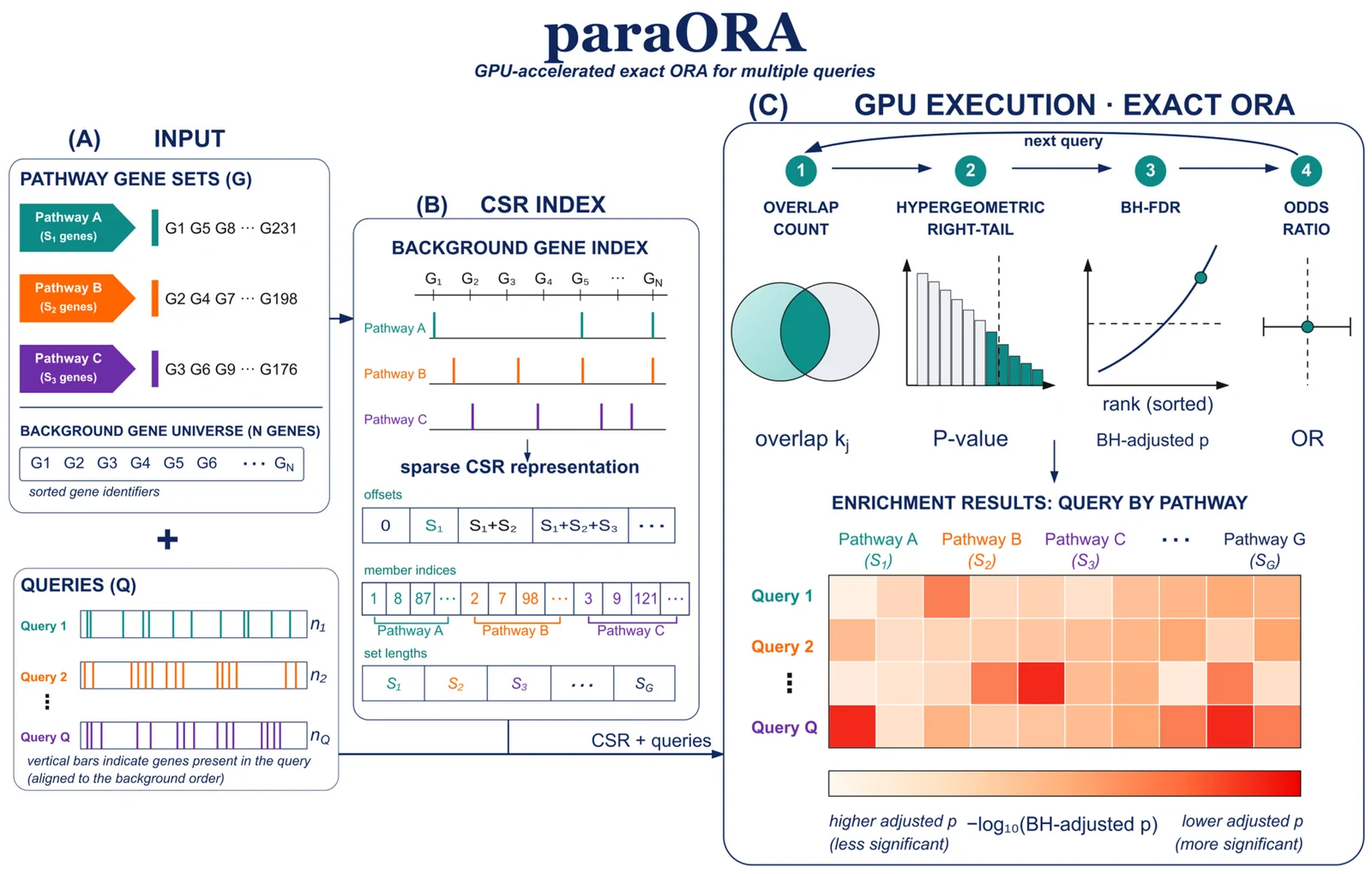
paraORA workflow for repeated exact ORA. The library is prepared once under an explicit background and reused for subsequent queries, with each gene list filtered and tested within the same gene universe. The CPU and GPU paths use the same ORA definition, whereas the GPU path keeps the main numerical stages on the device before returning a standardized enrichment table. In the schematic, *N* denotes background size, *S* gene-set size, *n* selected-list size, *k* overlap, *Q* the number of queries, and *G* the number of gene sets. The same notation is used in Equation (1).

For each contrast, the output reports the term identifier and description, background-filtered set size, overlap count, one-sided *p*-value, Benjamini–Hochberg (BH)-adjusted value, odds ratio, effective background size, retained selected-gene count, and actual execution path. Unless otherwise specified, multiple-testing correction is performed separately within each contrast. Figure 1 summarizes the workflow from input indexing to result output.

### 2.2. Reusable Gene-Set Library

During initialization, paraORA builds a shared integer index for background genes and gene-set members so that the GMT file does not need to be processed again for each query. Each gene set retains its effective members and size after background filtering. Memberships are stored in compressed sparse-row (CSR) form: a flat array contains all valid member indices, offsets delimit individual gene sets, and a length array records the filtered size of each set. If *E* is the number of valid membership links, *G* the number of gene sets, and *N* the background size, storage scales as *O*(*E* + *G* + *N*). For each query, the selected-gene list is mapped to a Boolean mask over the background [23], and the overlap for each gene set is obtained by summing mask values at its indexed members. Once initialized, the same library representation can be reused by either the CPU or GPU backend, separating one-time library preparation from query-specific computation.

### 2.3. Exact Inference and Multiple Testing

Once the background and overlap counts have been defined, paraORA uses a one-sided exact hypergeometric test to calculate the probability of observing at least *k* selected genes in a gene set under the specified background. The CPU reference calls scipy.stats.hypergeom.sf(*k* − 1, *N*, *S*, *n*) [24]. All main analyses use this exact test; no normal or other approximation is introduced, and no ranked-GSEA statistic is involved. Here, *N* is the background size, *S* is the background-filtered gene-set size, *n* is the retained selected-list size, and *k* is the observed overlap.

(1) P(X ≥ k)=∑x=max(k,n+S−N)min(S,n)SxN−Sn−xNn

Using CuPy for GPU array operations [25], the GPU path evaluates the same exact upper-tail probability on the device. It takes integer arrays of overlap counts and set sizes, evaluates log-combination terms in float64, and sums the shorter valid side of the hypergeometric distribution for each gene-set segment using a max-shifted log-sum-exp calculation. When the lower tail is shorter, the upper tail is obtained with a numerically stable expm1-based complement. Physical contingency-table bounds are checked before numerical evaluation. The GPU implementation therefore changes the execution strategy without changing the statistical definition.

During GPU-exact execution, overlap counts remain on the device and are passed directly to the exact hypergeometric test, BH correction, and odds-ratio calculation. The result arrays are copied back to CPU memory only after these numerical stages have been completed, after which they are assembled into the standard result table. Multiple contrasts are currently processed serially on a single GPU.

All *p*-values are adjusted within the specified testing scope using the BH procedure to control the false discovery rate (FDR) [26]. Odds ratios are calculated from the corresponding 2 × 2 contingency tables, with deterministic handling of zero-denominator boundary cases. The CPU and GPU paths use the same FDR scope and result-ordering rules. For cells *a* = *k*, *b* = *n* − *k*, *c* = *S* − *k*, and *d* = *N* − *n* − *S* + *k*, the ordinary odds ratio is *ad*/(*bc*). The implementation returns infinity when *bc* = 0 and *a* > 0, and 1 when *bc* = 0 and *a* = 0. When *bc* > 0 and *a* = 0, it returns 0 if *d* > 0 and 1 otherwise. Values of 1 in these degenerate cases are reporting conventions and should not be interpreted as evidence of a measured null effect.

### 2.4. Execution Backends

paraORA provides CPU, CPU-vectorized, GPU-hybrid, GPU-exact, and auto execution paths. The CPU path serves as the reference implementation. GPU-hybrid calculates overlap counts on the GPU and transfers them to the CPU for exact testing and FDR correction. GPU-exact keeps overlap counting, exact hypergeometric testing, FDR correction, and odds-ratio calculation on the GPU. The auto mode selects a GPU path when CUDA is available and otherwise uses the CPU path, while recording the backend actually used. Table 1 summarizes the execution paths.

### 2.5. GSE19429 Data Processing and Validation Setup

We reanalyzed the GSE19429 expression matrix distributed with rapidGSEA [16], derived from a study of bone-marrow CD34+ cells from 183 patients with myelodysplastic syndromes (MDS) and 17 healthy controls [27]. The repository matrix contains 20,639 gene identifiers. Its conversion script maps each probe to the first listed gene symbol and, for probes assigned to the same gene, retains the per-sample maximum value. The GEO sample record describes RMA-normalized signal intensities, and the repository conversion does not apply a logarithmic transformation. We therefore analyzed log2-transformed positive intensities without a pseudocount. This approach preserves the repository’s processed gene representation rather than reprocessing the original CEL files.

Differential expression was assessed with limma 3.66.0 in R 4.5.2 [28] using a two-group linear model and empirical-Bayes moderated *t*-tests with an abundance-dependent variance trend (eBayes, trend = TRUE). The contrast was defined as MDS minus control, with no clinical or technical covariates. BH adjustment was applied across all 20,639 tested genes. Genes were selected at adjusted *p* ≤ 0.05 and an absolute difference in mean log2 expression ≥ 1, and upregulated and downregulated genes were analyzed separately. Each ORA used all 20,639 tested identifiers as the explicit background and the 50 Hallmark sets from MSigDB v5.0, with effective set sizes of 1–5000 and BH adjustment across all 50 terms within each list. This limma-based case study used paraORA CPU; its tail probabilities and BH-adjusted values were cross-checked against R phyper and p.adjust using the same contingency counts.

The same limma-derived lists were also evaluated with clusterProfiler 4.18.4 in R 4.5.2. The background and Hallmark memberships were unchanged, and the full background was forced. In this version, enricher counts only query genes mapped to the supplied annotations, reducing the effective query sizes from 13 and 69 to 6 and 29. To preserve the complete query sizes, we added an auxiliary set containing all background genes and excluded it through the 5000-gene upper size limit; no Hallmark membership was altered. Because enricher returns only terms with positive overlaps, we restored zero-overlap Hallmark terms with *p* = 1 and applied R p.adjust across all 50 terms. We retained both the native results under the forced background and the results under these aligned statistical settings in the Supplementary Validation Addendum.

A separate implementation comparison used the fixed 500-gene query ranked by mean expression difference in the original source units. paraORA CPU, GPU-exact, and clusterProfiler 4.20.0 analyzed the same query, explicit background, and Hallmark mapping. clusterProfiler enricher was run with a forced universe, minimum and maximum set sizes of 1 and 5000, cutoffs of 1, and the full result slot. This comparison is separate from the matched-definition clusterProfiler validation of the limma-derived lists. GPU-exact validation of the biological inputs remains limited to the fixed top-500 query.

### 2.6. Numerical Validation and Repeatability

We evaluated numerical closeness, inferential agreement, and repeatability as separate criteria. The expanded validation matrix contained 425 valid contingency-table cases spanning four background sizes, 14 selected-list regimes, and gene sets ranging from empty and singleton sets to sparse, moderate, dense, and full-background cases. GPU-exact was compared with scalar SciPy on RTX 4090, RTX 4060, and NVIDIA A40 GPUs (NVIDIA Corporation, Santa Clara, CA, USA). Numerical closeness required |test − reference| ≤ 1 × 10^−12^ + 1 × 10^−10^ |reference| for each *p*-value and BH-adjusted value. Inferential agreement was evaluated at decision thresholds of 0.01, 0.05, and 0.10, together with term-aligned ranks, Top-10/20/100 membership, and significant-term counts. The earlier six full-library comparisons used rtol = 2 × 10^−10^ and atol = 1 × 10^−12^ and are reported separately in Table S1. Repeatability was assessed over 100 consecutive runs on a fixed input. The fixed-input repeatability results are provided in Table S2. In the separate shared-resource experiment, rapidGSEA GPU timing was conditional on enrichment-score agreement (Table S3), and CPU/GPU timings were compared within each workflow and preparation boundary (Table S4). The aggregate performance library contains 141,002 gene sets (Table S5). Expanded numerical results are reported separately from the real-library timing sentinels because these checks use different inputs and validation scopes (Tables S6 and S7).

Automated tests covered input normalization, explicit-background handling, CSR construction, statistical boundary cases, backend routing, multi-contrast analysis, benchmark contracts, result assembly, and the Web interface. The focused edge-case contract suite passed seven tests. Numerical and full-backend validation results are reported in the accompanying validation records. Windows was also used for the archived RTX 4060 Laptop GPU numerical evaluation and stage profile; environment records are reported separately for each experiment.

### 2.7. Performance Workloads and Timing

The primary repeated-query experiment measured complete in-memory workflows, with library initialization and subsequent query execution recorded separately. The benchmark used 141,002 gene sets, a 35,158-gene background, and 500-gene selected lists. Repeated-query scaling was evaluated with 100 deterministic, distinct lists at 1, 5, 10, 25, 50, and 100 serial queries, with one warm-up followed by five timed repetitions. The query-stage boundary included normalization, mask construction, overlap counting, exact hypergeometric testing, within-query BH-FDR, odds-ratio calculation, device-to-host transfer, and result-frame construction; file writing was measured separately. The resulting 1–100-query series therefore represents a parse-once, complete in-memory workflow rather than a file-to-file workflow. A separate prepared-library diagnostic compared complete query calls across execution backends while excluding parsing, library construction, and device initialization (Table S8).

A benchmark-only batched path was also evaluated on the RTX 4090 using batch sizes of 1, 4, 8, 16, 32, and 100. The real aggregate library combines Enrichr, MSigDB, and project-derived single-cell gene sets, including 142 retained mouse-labeled MSigDB sets. It was used as a deterministic computational workload rather than a species-pure interpretation library. Retained set sizes ranged from 3 to 1978; the biological case study instead used the specified Hallmark collection.

An independent eight-stage profile measured GMT parsing, library preparation, host-to-device transfer, overlap counting, hypergeometric testing, BH correction, result assembly, and file writing on the real 141,002-set library. Stage shares were calculated from the sum of the eight stage medians. This profile represents a different workload from the synthetic matched-tool experiment described below. We also recorded device-level sampled memory use for the 100-query workload and audited the deployed web-service settings.

Matched file-to-file comparisons used deterministic synthetic libraries containing 1000, 5000, 10,000, 50,000, 100,000, and 141,002 gene sets, with a 20,000-gene background, a 500-gene query, and 32 members per set. paraORA (CPU), paraORA (GPU-exact), GSEApy 1.3.1 (CPU), and the GOATOOLS 1.6.5 Fisher engine received identical inputs and wrote standardized term, overlap, set-size, *p*-value, and BH-FDR fields. GOATOOLS used its one-sided greater Fisher test without ontology propagation. Each measurement consisted of one warm-up followed by five repetitions with rotating tool order, one CPU thread, and explicit GPU synchronization. Timing covered input parsing through TSV output but excluded process startup, imports, and CUDA-context creation. Results were aligned by term before numerical and threshold-decision comparisons. Matched workflow medians are reported in Table S9. The best observed configurations from the separate batch experiment are summarized in Table S10.

### 2.8. Hardware and Software Environment

Primary performance measurements were performed on a Linux system with an RTX 4090 GPU, an Intel Core i9-13900K CPU (Intel Corporation, Santa Clara, CA, USA; 32 logical processors in the recorded host inventory), and approximately 126 GiB of system memory. The matched-tool inventory listed two RTX 4090 devices, but the benchmark exposed and used only device 0 (24,564 MiB). The recorded software environment was Python 3.12.3, NumPy 2.5.1, SciPy 1.18.0, pandas 3.0.5, CuPy 14.1.1, CUDA runtime 12.9, and NVIDIA driver 595.84. CPU comparisons used one thread through the recorded OpenMP, MKL, OpenBLAS, and NumExpr settings. GPU probabilities, BH-adjusted values, and odds ratios were calculated in float64. Environment records are experiment-specific; these versions are not assigned by default to the prepared-backend diagnostic or biological reanalysis. RTX 4060 Laptop and A40 GPUs were also included in the numerical evaluation; performance conclusions remain specific to the measured workloads and hosts. Experiment-specific software records are provided in Table S11. For a reproducible single-thread baseline, set OMP_NUM_THREADS = 1, MKL_NUM_THREADS = 1, OPENBLAS_NUM_THREADS = 1, and NUMEXPR_NUM_THREADS = 1 before starting Python. Without explicit settings, host-library thread behavior depends on the installed libraries and environment; the usage guide provides Linux and PowerShell commands.

Additional archived measurements comprised eight-stage profiles on an RTX 4060 Laptop GPU and an NVIDIA A40, and the same 1–100-query protocol on the A40, each with five timed repetitions after warm-up. The A40 host used an Intel Xeon Gold 6148 CPU and Linux; the RTX 4060 Laptop GPU was evaluated on Windows. Their environment records did not explicitly set the OpenMP, MKL, OpenBLAS, or NumExpr thread variables. These measurements therefore provide additional within-platform evidence, rather than a controlled comparison of GPU throughput across hosts. Their timing and environment records are provided in the Supplementary Validation Addendum.

### 2.9. Software Use and Availability

The paraORA v0.1.0 local interface accepts selected-gene lists, an explicit background, and a GMT library, and returns term-level statistics together with the backend actually used. Supplementary Note S1 provides a minimal command, identifier-handling rules, and the audited web-service limits. The public service accepts bounded jobs, while local execution gives users direct control over data handling and computational resources. Deployment observations reported in the Supplementary Materials are dated and should not be interpreted as permanent service guarantees. The GitHub documentation provides installation and thread-setting instructions in docs/usage.md, a minimal CLI example, and a reusable-library example in examples/reuse_library.py; docs/reproducibility.md maps each analysis to its inputs, script, and archived output.

## 3. Results

### 3.1. Numerical Agreement Across GPU Models

GPU-exact met the prespecified combined absolute-and-relative tolerance for all 425 *p*-values and BH-adjusted values on each of the three tested GPU models. The maximum absolute differences were 3.72 × 10^−11^ for *p*-values and 2.89 × 10^−11^ for BH-adjusted values. Threshold decisions, term-aligned ranks, Top-10/20/100 membership, and significant-term counts were unchanged. The vectorized CPU comparator met the same criterion in 424/425 *p*-value cases and 425/425 BH-adjusted-value cases. Its single *p*-value exception had a normalized error of 1.006, without changing the evaluated inference. These comparisons show numerical agreement on the tested inputs, not bitwise identity.

**Figure 2:**
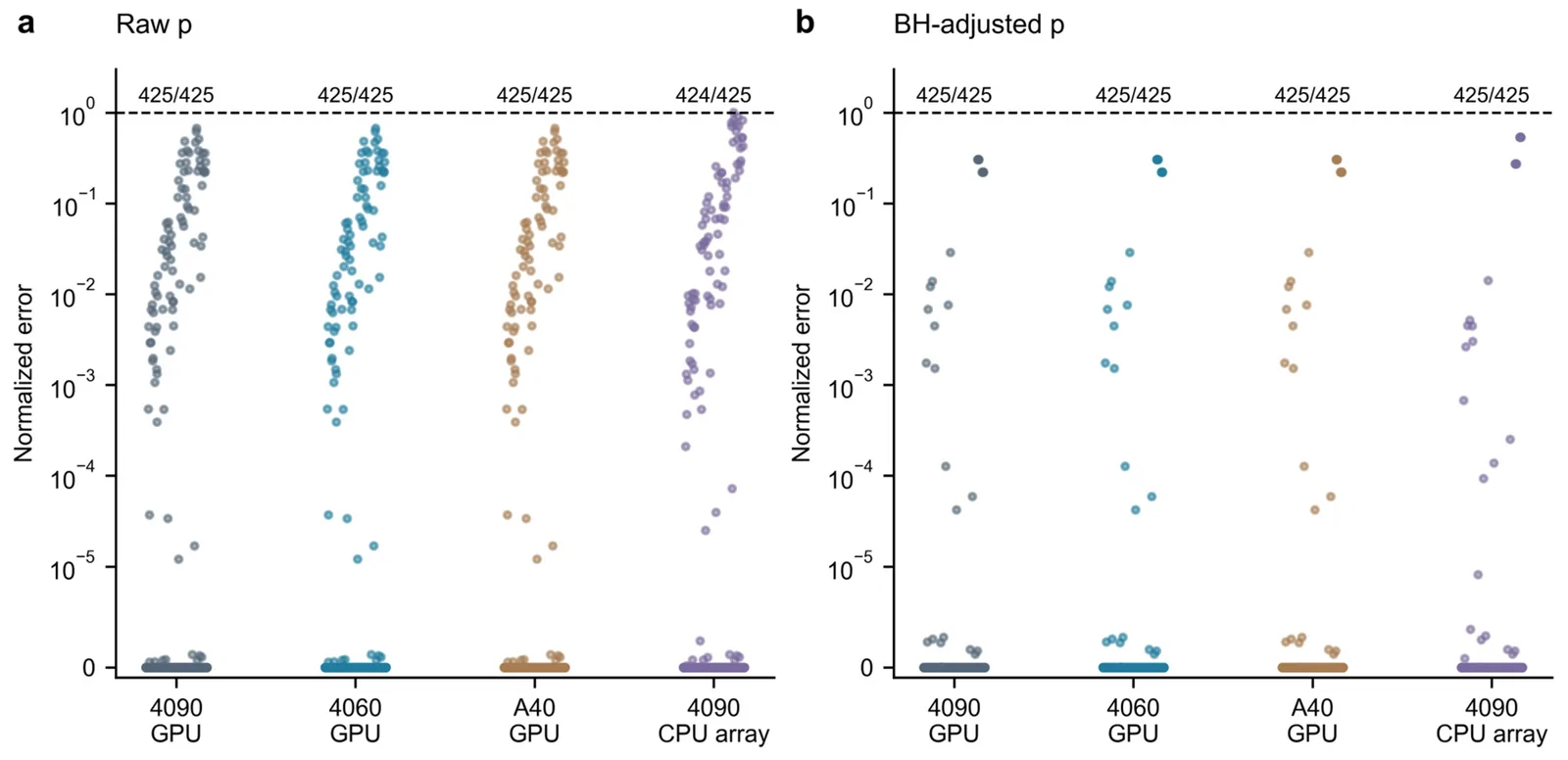
Numerical agreement in the 425-case matrix. (**a**) Raw *p*-values. (**b**) BH-adjusted *p*-values. Each point represents one case; horizontal offsets follow source order and are included only for visibility. The ordinate is |candidate − scalar SciPy reference|/(10^−12^ + 10^−10^ |reference|). The dashed boundary at 1 marks the combined acceptance criterion. A symmetric-log scale includes exact zero, with a linear interval extending to 10^−5^. Labels indicate the number of passing cases. GPU-exact passes 425/425 cases on each GPU model for both metrics, whereas the RTX 4090 CPU-array comparator passes 424/425 raw-*p* comparisons and 425/425 BH comparisons. Device labels indicate the evaluation environments and should not be interpreted as cross-host performance rankings.

Figure 2 and Table 2 summarize the numerical comparisons. In separate large-library sentinel checks from the repeated-query and stage-profile experiments, the raw *p*-values did not meet the specified tolerance, with a maximum absolute difference of 8.72 × 10^−11^, whereas the BH-adjusted values and tested FDR decisions agreed. These sentinel checks were not designed to establish numerical closeness for every query in the timing series.

### 3.2. Biological Case Study and External Validation

**Figure 3:**
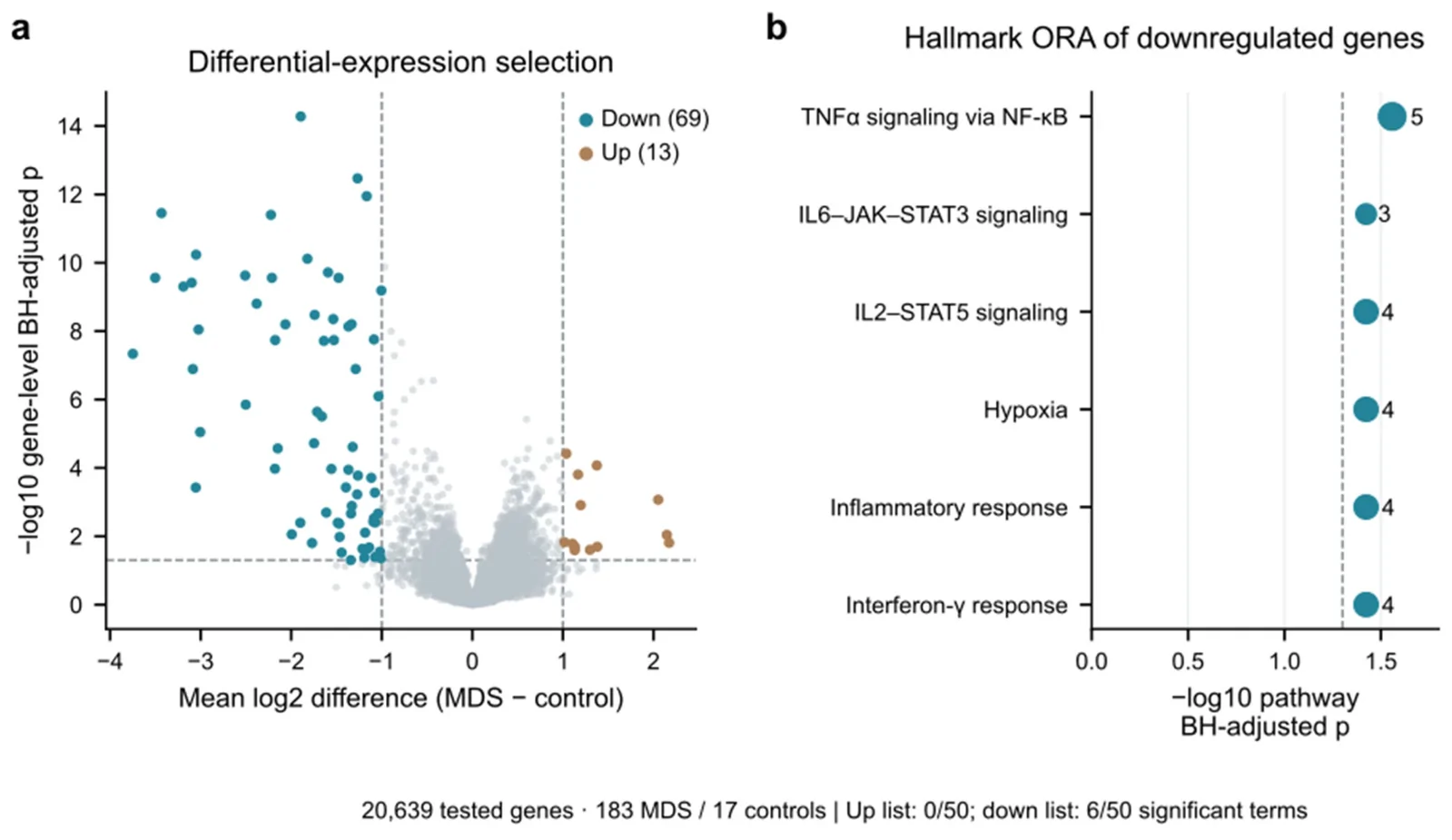
Hallmark ORA following an exploratory limma reanalysis of GSE19429. (**a**) All 20,639 genes tested in 183 MDS and 17 control samples. The horizontal dashed line indicates a gene-level BH-adjusted *p* = 0.05, and the vertical dashed lines indicate mean log2-expression differences of −1 and +1. Gray points represent genes that failed at least one of these two thresholds and were not included in the selected upregulated or downregulated gene lists. Colored points meet both criteria: 69 genes were downregulated and 13 were upregulated. (**b**) All six significant Hallmark terms from the downregulated list; dot areas and numeric labels indicate overlap counts. Pathway-level BH adjustment includes all 50 terms tested within each list. No significant terms were identified in the upregulated list. The analysis uses the archived gene-level intensity matrix, a two-group limma model without covariates, and paraORA CPU.

The limma analysis selected 13 upregulated and 69 downregulated genes (Figure 3a). Hallmark ORA identified six terms with BH-adjusted *p* ≤ 0.05 in the downregulated list and none in the upregulated list. The six downregulated-list terms were TNFα signaling via NF-κB, IL6–JAK–STAT3 signaling, IL2–STAT5 signaling, hypoxia, inflammatory response, and interferon-γ response (Figure 3b; Table S12). TNFα signaling via NF-κB contained five selected genes among 200 background-filtered members (*p* = 5.53 × 10^−4^; BH-adjusted *p* = 0.0276). The other five terms had adjusted *p* = 0.0377, with three or four overlapping genes.

The selected genes were over-represented in immune-response and signaling annotations in the measured CD34+ population. For the downregulated list, this enrichment indicates that its members occur more often than expected in these annotations; it does not directly measure pathway activity. The unequal sizes of the two selected-gene lists and the sharing of genes across Hallmark sets also influence the observed pattern. The analysis therefore treats the upregulated and downregulated lists separately under the same background.

The same limma lists were additionally checked against clusterProfiler under the aligned full-query and 50-term BH definitions. Across all 50 terms in each direction, overlap counts and decisions at BH-adjusted *p* ≤ 0.05 agreed with paraORA CPU. Maximum absolute differences were 5.00 × 10^−16^ for *p*-values and 2.33 × 10^−15^ for BH-adjusted values, retaining zero significant terms in the upregulated list and six in the downregulated list. With the full background forced but clusterProfiler’s native query filtering and BH scope retained, the corresponding counts were five and twelve. This difference reflects the analysis definitions and does not indicate numerical disagreement under matched settings (Supplementary Validation Addendum).

For the limma-derived lists, the maximum differences from R phyper were 5.00 × 10^−16^ for *p*-values and 2.33 × 10^−15^ for BH-adjusted values. In the separate fixed top-500 comparison (Table S13), all 50 term identifiers, overlap counts, and effective set sizes matched those from clusterProfiler, with 29 significant terms. Maximum absolute differences from clusterProfiler were 9.44 × 10^−16^ for *p*-values and 1.11 × 10^−15^ for BH-adjusted values with paraORA CPU, and 1.98 × 10^−11^ and 2.24 × 10^−11^, respectively, with GPU-exact. Threshold decisions agreed at 0.01, 0.05, and 0.10. The fixed top-500 comparison and the limma-list validations use different inputs and retain separate validation scopes and software-version records.

### 3.3. Repeated-Query Performance

**Figure 4:**
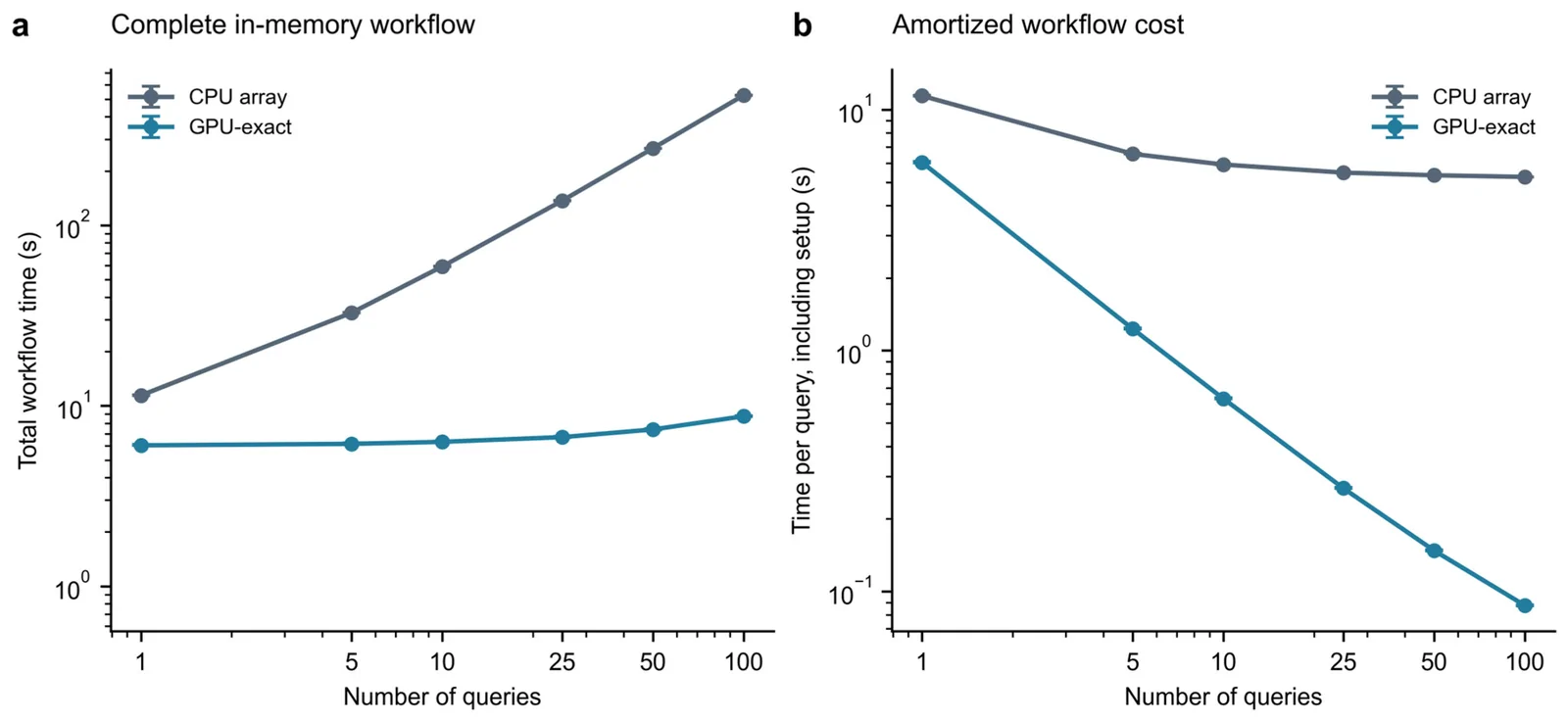
Complete repeated-query performance on RTX 4090. (**a**) Total in-memory workflow time. (**b**) Total workflow time divided by the number of queries. Points show medians, and error bars indicate the 25th–75th percentiles from five complete repetitions at each checkpoint (1, 5, 10, 25, 50, and 100 queries). The aggregate library contains 141,002 gene sets and 35,158 background genes; each selected list contains 500 genes. Workflow timing includes one-time setup and complete query execution but excludes per-query file writing. The reduction in amortized cost reflects the sharing of one-time costs and does not mean that the statistical calculation itself becomes faster as queries accumulate.

The median complete in-memory runtime was 11.418 s on CPU and 6.037 s on GPU for one query, compared with 525.792 s on CPU and 8.749 s on GPU for 100 serial queries. The corresponding CPU/GPU runtime ratio increased from 1.89 to 60.10. Because one-time parsing and preparation costs were shared across queries, the average workflow time per query decreased as the number of queries increased. Each query nevertheless required its own overlap counting, hypergeometric testing, and BH correction. In a separate benchmark, the fastest tested batch configuration reduced elapsed time by 2.80–4.93% relative to its serial baseline. Reusing a prepared library therefore provided increasing practical benefit across repeated queries, whereas the tested batching extension produced only a modest additional gain. Figure 4 shows the total and amortized workflow times. Complete checkpoint timings are provided in Table S14.

The archived A40 experiment showed the same benefit of sharing preparation costs: median CPU/GPU workflow times were 31.267/13.619 s for one query and 1685.703/19.426 s for 100 queries, giving within-host ratios of 2.30 and 86.78, respectively. These timings include preparation and exclude file writing. Host and software differences prevent interpreting the A40 and RTX 4090 ratios as a ranking of the GPUs (Supplementary Validation Addendum).

### 3.4. Complete-Workflow Performance and Runtime Costs

**Figure 5:**
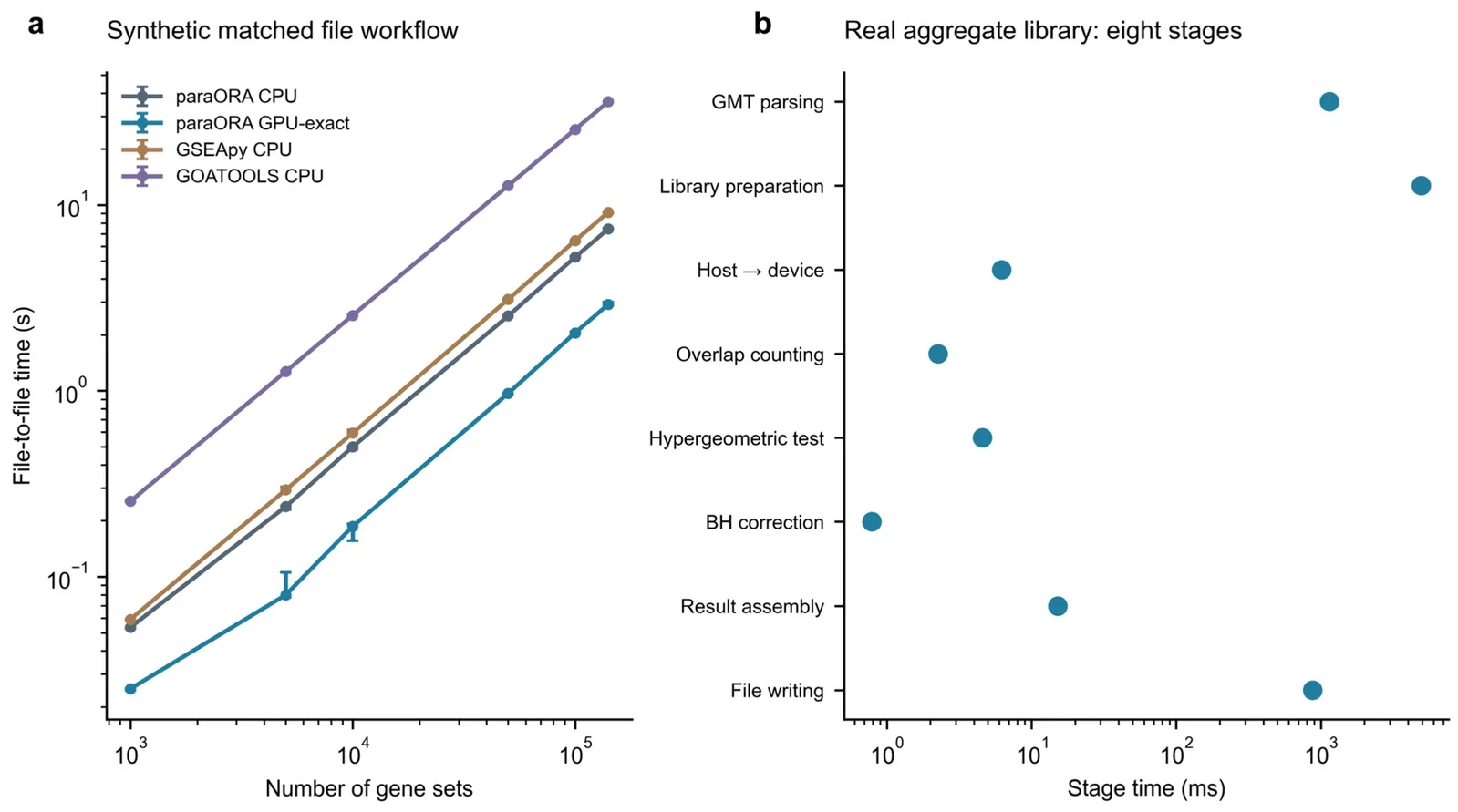
Complete-workflow costs from two independent experiments on RTX 4090. (**a**) Matched synthetic file-to-file benchmark with 20,000 background genes, 500 selected genes, and 32 genes per set across all six measured library sizes. All tools use the same normalized output contract; GOATOOLS denotes the flat-GMT Fisher-engine adapter. (**b**) Eight measured stages for the real aggregate library containing 141,002 gene sets, 35,158 background genes, and 500 selected genes. Points and error bars show medians and 25th–75th percentiles from five technical repetitions. The stage measurements in (**b**) do not decompose the runtimes in (**a**), and the sum of stage medians is not equivalent to the median total runtime.

In the synthetic matched-tool experiment, GPU-exact was faster than paraORA CPU at all six tested library sizes. At 141,002 gene sets, the median file-to-file runtime was 2917.8 ms for GPU-exact, compared with 7432.1 ms for paraORA CPU, 9140.7 ms for GSEApy, and 35,965.5 ms for the GOATOOLS flat Fisher engine. GPU-exact was therefore 2.55-fold faster than paraORA CPU at this scale. All 24 tool-by-scale comparisons passed the recorded numerical and inferential checks. These comparisons apply to the explicitly matched flat gene-set task and do not evaluate ontology-aware GO analysis. Figure 5a shows the matched comparison, and Table 3 summarizes the timing boundaries.

In the independent eight-stage profile of the real aggregate library, library preparation, GMT parsing, and file writing accounted for 70.6%, 16.4%, and 12.6%, respectively, of the sum of the stage medians. These measurements show that substantial workflow time can remain outside GPU numerical execution. The synthetic matched-tool comparison and real-library stage profile used different backgrounds and set-size distributions, so their absolute runtimes and stage shares are not directly interchangeable. Figure 5b shows the stage-level measurements. The stage medians and dispersion are provided in Table S15.

The archived RTX 4060 Laptop and A40 stage profiles likewise showed that library preparation dominated the sum of stage medians, accounting for 76.8% and 74.8%, respectively. The RTX 4060 overlap-counting stage varied from 15.413 to 262.707 ms across five repetitions, so its dispersion is retained alongside the median. Both profiles retained the real-library raw-*p* tolerance failures described in Section 3.1, while overlap counts, BH-adjusted values and tested decisions agreed (Supplementary Validation Addendum).

In a separate prepared-library diagnostic, median query times were 5277.220 ms for vectorized CPU, 8047.872 ms for GPU-hybrid, and 33.076 ms for GPU-exact. The recorded hypergeometric component of the hybrid path was 8002.111 ms, indicating that CPU-side probability evaluation dominated this execution path. Because the hybrid and vectorized CPU paths use different CPU execution strategies, the difference between their runtimes cannot be attributed solely to host–device transfer. Backend medians are reported independently of component sums. The diagnostic used 500 selected genes and a 35,158-gene background and excluded parsing, library construction, and device initialization.

## 4. Discussion

paraORA is designed for studies that interpret multiple selected-gene lists against the same annotations. By fixing the background and reusing a prepared gene-set index, repeated queries can be analyzed under the same statistical definition while retaining separate tests and multiple-testing correction for each list. The GSE19429 reanalysis illustrates this use case with independently analyzed upregulated and downregulated gene lists derived from an explicit selection procedure.

For the evaluated inputs, GPU execution reproduced the CPU-reference overlap counts and downstream inference while substantially reducing query time once the library had been prepared. The advantage was smaller when parsing, preprocessing, and file writing were included, showing that end-to-end performance depends on how much of the workflow is spent on repeated query computation. In the separate batching diagnostic, only overlap counting is batched; exact testing, BH correction, odds-ratio calculation, result assembly, and the timed sorting and signature checks remain query-specific. This limited batching scope helps explain the modest 2.80–4.93% runtime reduction, without identifying a hardware bottleneck from these measurements.

Numerical agreement should be interpreted together with the inferential outputs of interest. The expanded validation matrix met the prespecified GPU tolerance on all three GPU models, and the fixed top-500 clusterProfiler comparison produced the same tested FDR decisions. At the same time, the vectorized CPU exception in the validation matrix and the raw-*p* sentinel failures in the large-library experiments show that numerical closeness and inferential agreement are distinct criteria. An exact statistical definition therefore does not imply bitwise equality across implementations. The same-limma comparison further shows why an identical gene-list file alone does not ensure a matched ORA analysis: effective query size and the multiple-testing family must also be aligned.

The appropriate enrichment method depends on the form of the biological input. rapidGSEA and fgsea operate on ranked lists, whereas paraORA performs ORA on selected-gene lists under an explicit background. Resources and software such as clusterProfiler provide broader annotation and enrichment workflows. paraORA contributes a reusable library representation and GPU-based numerical execution for repeated exact ORA; it does not introduce a new enrichment statistic.

Several limitations define the scope of the current evaluation. Numerical testing included RTX 4090 and RTX 4060 GPUs based on the Ada architecture and an A40 based on Ampere, while the primary performance measurements were obtained on an RTX 4090 system. Additional RTX 4060 Laptop and A40 timings broaden the measured hardware coverage, but differences in hosts, software and thread settings prevent a controlled cross-GPU performance comparison. Multiple contrasts remain serial in the production API, and the separate batching benchmark reduced elapsed time by only 2.80–4.93%. The timing experiments also exclude different initialization costs according to their respective boundaries in Table 3. The recorded 2405 MiB device-memory value is a sampled peak and may not capture brief allocations. In the biological case study, the archived processing retains historical probe pooling and gene identifiers, and the differential-expression model contains no clinical or technical covariates. Disease heterogeneity and unmodeled clinical or technical factors may therefore affect the selected-gene lists. The analysis is intended as an exploratory software use case and does not establish a replicated MDS expression signature or a clinical mechanism.

## 5. Conclusions

paraORA implements exact one-sided ORA for repeated selected-gene lists analyzed against a fixed explicit background and gene-set library. GPU-exact preserved the evaluated inferential outputs across the three tested GPU models. On the RTX 4090 workload, the practical speedup increased as more queries shared the one-time preparation cost, whereas complete file-to-file acceleration remained constrained by library preparation and output. paraORA therefore provides a reusable implementation for repeated ORA when consistent backgrounds, gene-set definitions, and statistical procedures are required across queries.

## Figures and Tables

**Table 1:** Execution paths. GPU-exact and CPU use the same ORA definition and differ in their numerical implementation and execution location. Final result arrays are returned to the CPU for table assembly. Multiple contrasts are currently processed serially on one GPU.

Execution Path	Overlap Counting	Exact Inference and BH-FDR	Result Assembly
Scalar CPU reference	CPU	CPU (scalar SciPy)	CPU
Vectorized CPU	CPU	CPU (array SciPy)	CPU
Hybrid GPU diagnostic	GPU	CPU	CPU
GPU-exact ORA	GPU	GPU	CPU

**Table 2:** Numerical validation. Comparisons were aligned by term identifier, and rank ties were resolved according to the original term order. Top-*k* denotes membership at *k* = 10, 20, and 100. The repeatability audit showed no changes in FDR decisions, ranks, Top-*k* membership, or significant-term count.

Comparison	Evaluation Set	Max *p*-Value Difference	Max BH-FDR Difference	Outcome
Vectorized CPU vs. scalar SciPy	425 cases	5.62 × 10^−11^	5.06 × 10^−11^	*p*: 424/425; BH: 425/425; inference unchanged
GPU-exact vs. scalar SciPy	425 cases × 3 GPU models	3.72 × 10^−11^	2.89 × 10^−11^	425/425; criterion met

**Table 3:** Performance workloads and timing boundaries. Each experiment uses its own repetition scheme and initialization boundary. Detailed medians and dispersion are provided in the Supplementary Tables.

Experiment	Background/Library	Queries	Measured Boundary	Key Exclusions
Repeated queries	35,158 genes; 141,002 real aggregate sets	1–100 distinct lists; 500 genes each	Parse once, prepare once, complete in-memory results	Per-query file writing
Eight-stage profile	35,158 genes; 141,002 real aggregate sets	One 500-gene list	Parsing through output, measured by stage	Process startup and imports
Matched-tool comparison	20,000 genes; 1000–141,002 synthetic sets; 32 members/set	One 500-gene list	Parsing through normalized TSV output	Startup, imports, CUDA context
Prepared-backend diagnostic	35,158 genes; 141,002 real aggregate sets	One 500-gene list	Complete query on prepared library	Parsing, preparation, device initialization

## Data Availability

The data supporting this study, including differential-expression results, enrichment outputs, benchmark measurements, and validation records, are provided in the Supplementary Materials and accompanying Supplementary Data. The biological case study uses publicly available data from GEO accession GSE19429. The paraORA web service is available at https://paraora.deeptools.top/ (accessed on 1 September 2026). The paraORA source code, usage examples, and selected numerical and benchmark results are available at https://github.com/rainlove1101/paraORA (accessed on 10 September 2026). The GitHub repository contains the full computational pipeline needed for the documented local workflows, including the offline CLI, reusable-library example, test suite, synthetic-data generators, analysis scripts, and provenance records described in the reproducibility guide. After installation and provision of the documented input files, these local workflows execute independently of the hosted paraORA service and other enrichment Web APIs. Third-party annotation libraries and expression matrices must be obtained under their provider terms. We commit to maintaining paraORA for at least five years from publication, including reproducible bug fixes, documentation updates, and dependency compatibility guidance.

## Supplementary Materials

The following supporting information can be downloaded at: https://www.mdpi.com/article/10.3390/biology15181641/s1, Figure S1, Tables S1–S15, and Supplementary Note S1 accompany this manuscript. Supplementary Data contains gene-level and ORA results, benchmark measurements, source records, and a file guide. The Supplementary Validation Addendum provides the same-limma clusterProfiler inputs, native and aligned outputs, comparison tables, reproducible script and software versions, together with archived RTX 4060 Laptop and A40 timing records.

## Abbreviations

The following abbreviations are used in this manuscript:

BH	Benjamini–Hochberg
CPU	Central processing unit
CSR	Compressed sparse row
CUDA	Compute Unified Device Architecture
DEG	Differentially expressed gene
ES	Enrichment score
FDR	False discovery rate
GEO	Gene Expression Omnibus
GMT	Gene Matrix Transposed
GO	Gene Ontology
GPU	Graphics processing unit
GSEA	Gene set enrichment analysis
IQR	Interquartile range
MDS	Myelodysplastic syndromes
MSigDB	Molecular Signatures Database
ORA	Over-representation analysis
RMA	Robust multi-array average
TSV	Tab-separated values
