# Supplementary material for: paraORA: GPU-Accelerated Exact Over-Representation Analysis for Repeated Queries of Large Gene-Set Libraries
Source: Biology (Basel). 2026 Sep 17;15(18):1641. doi: 10.3390/biology15181641 (PMC13604057; doi:10.3390/biology15181641)
Supplement: Supplementary file 1 [file biology-15-01641-s001.zip › paraORA_Biology_R2_Supplementary_Clean.pdf]

Supplementary Materials

paraORA: GPU-Accelerated Exact Over-Representation Analysis for Repeated Queries of Large Gene-Set Libraries

Zheng Wu, Zejun Zhang, Wenqing Feng, Jinlei Sun, Zhichun Liu, Guoqiang Wang and Yunqing Liu

This supplement provides supporting methods, numerical comparisons, biological-case results and performance measurements. Bibliographic numbers refer to the reference list in the main manuscript. The accompanying Supplementary_Data folder contains the tabular results and source records described below. The Supplementary Validation Addendum cited in the revised manuscript is integrated here and supplied in validation_addendum/ within Supplementary_Data. It adds same-limma external validation and archived RTX 4060 Laptop and A40 timing evidence without changing Tables S1–S15.

S1. Numerical agreement and repeatability

The six historical CPU–GPU comparisons assessed overlap counts, raw p-values, BH-adjusted p-values, FDR decisions, term-aligned ranks, Top-10/20/100 membership and significant-term counts (Table S1). Their original combined tolerance was atol = 1×10^{-12} and rtol = 2×10^{-10}. Repeatability used 100 consecutive GPU-exact runs on a fixed input. These are separate validation records; their tolerances are not retrospectively pooled.

Table S1. Numerical agreement between CPU and GPU-exact ORA.

Mode	Contrast	Max |delta p|	Max |delta BH-FDR|	Inference
Single	Liver endothelial	7.670e-11	1.084e-10	Unchanged
Single	Pancreas alpha	7.670e-11	1.252e-10	Unchanged
Single	PBMC B cell	7.977e-11	1.338e-10	Unchanged
Multi	Liver endothelial	7.670e-11	1.084e-10	Unchanged
Multi	Pancreas alpha	7.670e-11	1.252e-10	Unchanged
Multi	PBMC B cell	7.977e-11	1.338e-10	Unchanged

Note: All comparisons contained 141,002 gene sets. Overlap counts, FDR decisions, ranks, Top-10/20/100 membership, and significant-term counts were unchanged.

Table S2 summarizes 100 consecutive GPU-exact runs on the same prepared input.

Table S2. Repeatability of GPU-exact ORA in 100 fixed-input runs.

Repeatability metric	Value
Fixed-input GPU-exact repetitions	100
Maximum p-value range	4.44×10^{-16}
Maximum BH-FDR range	6.66×10^{-16}
FDR decisions (0.01, 0.05, 0.10)	0 changes
Ranks; Top-10/20/100; significant-term count	0 changes

BH-FDR denotes Benjamini–Hochberg-adjusted p-values; Top-k denotes membership among the k highest-ranked terms. Source data: numerical/historical_comparisons.tsv and numerical/repeatability_100_runs.tsv.

S2. Shared-resource rapidGSEA comparison

The shared-data comparison used the GSE19429 matrix and PATIENT/CONTROL labels distributed with the rapidGSEA repository. rapidGSEA was run as a complete ranked-GSEA workflow with 1,024 phenotype permutations. paraORA used the top 500 genes ranked by mean(PATIENT) minus

mean(CONTROL), with all 20,639 genes as the explicit background. CPU-to-GPU ratios are reported within each workflow only and are not cross-method speed comparisons. The top-500 list is an implementation-validation input selected by a mean-difference ranking, not a formal differential-expression gene list.

The expression data were distributed with rapidGSEA [16] and originated from GSE19429 [27]. Figure S1 presents within-workflow GPU acceleration; it does not compare ORA and GSEA as equivalent statistical tasks.

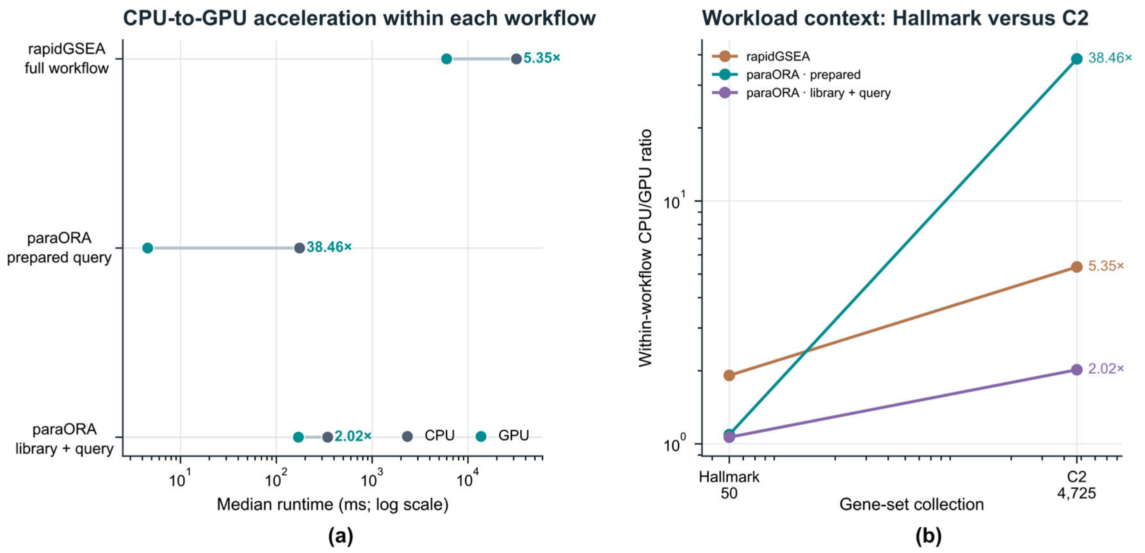

Figure S1. Within-workflow GPU acceleration using shared expression data. (a) CPU and GPU median runtimes for the C2 collection, with ratios calculated within each timing boundary. (b) Ratios for Hallmark (50 source sets) and C2 (4725 source sets). rapidGSEA uses a complete ranked-GSEA workflow; paraORA uses a derived 500-gene ORA query and the indicated preparation boundary. Ratios do not rank the two methods against each other.

Table S3 reports the original enrichment-score (ES) agreement gate used before rapidGSEA GPU timing.

Table S3. Numerical eligibility of rapidGSEA GPU timing on repository-bundled data.

Gene-set library / precision	Sets assessed	Maximum absolute original-ES difference	Gate	GPU timing decision
Hallmark / default	50	0	1×10^{-5}	Eligible
C2 / default single precision	4,725	4.8×10^{-5}	1×10^{-5}	Excluded
C2 / double precision	4,725	0	1×10^{-10}	Eligible

Note: Original enrichment-score (ES) agreement was checked before formal GPU timing. C2 timing in Supplementary Table S4 uses the eligible double-precision path.

Table S4 reports the separate timing boundaries. Both tools used one warm-up and five timed repetitions; all ratios compare CPU and GPU within the same workflow.

Table S4. CPU and GPU timing details for rapidGSEA and paraORA on shared data resources.

Library	Workflow and timing boundary	CPU median (ms)	GPU median (ms)	CPU range (ms)	GPU range (ms)	CPU/GPU ratio
Hallmark (50 sets)	rapidGSEA, full command line, default precision	1,110	580	1,080–1,140	560–580	1.91
Hallmark (50 sets)	paraORA, prepared library plus one 500-gene query	3.889	3.561	3.862–4.036	3.510–4.029	1.09
Hallmark (50 sets)	paraORA, library construction plus one 500-gene query	13.092	12.288	12.694–14.067	12.048–14.796	1.07
C2 (4,725 source sets)	rapidGSEA, full command line, double precision	32,050	5,990	31,790–32,140	5,950–6,000	5.35
C2 (4,725 source sets)	paraORA, prepared library plus one 500-gene query	175.001	4.550	171.553–180.038	4.478–4.932	38.46
C2 (4,725 source sets)	paraORA, library construction plus one 500-gene query	343.467	170.103	340.091–365.838	164.783–204.933	2.02

rapidGSEA directly reads the complete expression matrix and performs gene ranking, GSEA enrichment-score calculation, 1,024 phenotype permutations, and result output. For paraORA, genes were ranked by mean(PATIENT) minus mean(CONTROL); the top 500 genes formed the ORA query list, and all 20,639 gene identifiers formed the explicit background. paraORA then performed one-sided exact hypergeometric testing and BH-FDR correction. rapidGSEA timing covered its complete command-line workflow, whereas paraORA was measured at prepared-query and library-construction-plus-query boundaries. Both tools used one warm-up and five timed repetitions, reported as medians. rapidGSEA CPU/GPU agreement was checked using the original enrichment score (ES); the C2 default single-precision path exceeded the prespecified ES tolerance and was excluded from formal GPU timing, so the numerically concordant double-precision path was used.

The single-precision original-ES gate (10^{-5}) and the double-precision gate (10^{-10}) are evaluation thresholds for CPU–GPU ES agreement, not ORA p-value tolerances or statistical significance thresholds. C2 single precision failed its gate (4.8×10^{-5}) and its GPU timing was excluded. The source records do not establish that an ES tolerance guarantees identical permutation p-values, so no such equivalence is inferred. Within-workflow CPU/GPU ratios in Table S4 remain contextual measurements.

C2 contains 4725 source sets, of which 4718 were retained for the paraORA comparison. Raw timing summaries and ES gate records are supplied in rapidgsea/.

S3. Benchmark library and expanded numerical evaluation

Table S5 describes the real aggregate library used for computational performance measurements.

Table S5. Composition of the 141,002-set performance benchmark library.

Source label	Unique sets before filtering	Retained sets	Biological/source note
Enrichr	105,438	105,383	Human-targeted/unspecified aggregate labels
MSigDB	52,326	35,379	35,237 human-labelled and 142 mouse-labelled retained sets
WikiPathways	1,125	0	No set survived the benchmark background and size filters
Project-derived single-cell	240	240	Fixed project benchmark collection
Total	159,129	141,002	13,006,221 retained memberships

Note: The background is the stable union of the three benchmark background files (35,158 symbols). Gene sets were intersected with that background and retained at effective sizes 3–5,000. Retained size summary: minimum 3, first quartile 19, median 46, mean 92.2414, third quartile 95, maximum 1,978. GMT SHA-256: 0bdc1cbe6bd22de2209d6f169e383cc5f7dc3bb9ae53ae9bc1f8839d853a40f8. Background SHA-256: 1bbe81e34e97ff158e90b95df6271a0f743f626567305327a12c077698a88790. The mixed-source aggregate is a deterministic performance workload and is not used as a species-pure biological interpretation library.

The expanded numerical matrix covers 425 valid contingency-table cases, not 425 biological datasets. Each candidate is compared with scalar SciPy using $|\text{candidate} - \text{reference}| \leq \text{atol} + \text{rtol}\,|\text{reference}|$, with atol = 10^{-12} and rtol = 10^{-10}. A normalized error divides the absolute difference by the right-hand side; values above 1 fail. RTX 4090 and RTX 4060 are Ada Lovelace devices and A40 is Ampere: three GPU models spanning two microarchitectures. This validation does not rank performance across hosts. The expanded matrix uses rtol = 10^{-10}, whereas the earlier comparisons use rtol = 2×10^{-10}. Table S6 reports normalized errors.

Table S6. Expanded numerical matrix normalized errors.

Device	Candidate	Max normalized error (p)	Passing p-values	Max normalized error (BH)	Passing BH values
RTX 4090	GPU-exact	0.676137	425/425	0.305032	425/425
RTX 4060	GPU-exact	0.676135	425/425	0.305032	425/425
A40	GPU-exact	0.676137	425/425	0.305032	425/425
RTX 4090	CPU array	1.005784	424/425	0.535770	425/425

The CPU-array raw-p exception is retained rather than rounded into a pass. Numerical agreement does not mean bitwise identity. The table reports the evaluated matrix only; sentinel checks from the real-library timing runs have a different comparison scope.

Table S7 reports separate sentinel checks from the real-library timing experiments.

Table S7. Real-library agreement checks and sentinel scope.

Record	Raw p criterion	Max |Δp|	BH criterion	Scope
Eight-stage RTX 4090	Fail	8.71744×10^{-11}	Pass; max Δ = 0	141,002 rows
Repeated-query warm-up	Fail	8.71745×10^{-11}	Pass; max Δ = 0	Query 1
Repeated-query timed rep 0	Fail	8.71744×10^{-11}	Pass; max Δ = 0	Queries 1 and 100

These checks compare CPU and GPU outputs under the recorded 10^{-12} absolute and 10^{-10} relative tolerance. Term identifiers, overlaps, odds ratios and FDR decisions at 0.01, 0.05 and 0.10 agree. Sentinel checks do not constitute full result validation of every query in all five timed repetitions.

Sources: library/benchmark_library_summary.json, numerical/expanded_matrix.tsv, repeated_query/agreement_summary.json and eight_stage/agreement_summary.json.

S4. Prepared-library execution paths

Prepared-library backend measurements are retained separately from the complete repeated-query and file-workflow benchmarks. Each backend is measured on its complete query call; medians, IQRs and ranges summarize the archived repetitions. The hybrid path uses GPU overlap counting, scalar per-set SciPy hypergeometric calls and host BH correction. The CPU-array path uses an array SciPy call. Their difference cannot be attributed solely to data transfer or solely to the Python loop without a controlled component experiment.

Table S8 reports five repetitions of the complete prepared-query call on RTX 4090, using 141002 retained sets, a 35158-gene background and 500 selected genes; parsing, library preparation and device initialization were excluded.

Table S8. Complete prepared-query backend timings.

Backend	Median ms	IQR ms	Range ms	CPU / backend
cpu-vectorized	5277.220	11.632	5264.964–5286.079	1.000
gpu-hybrid	8047.872	61.761	8002.045–8115.884	0.656
gpu-exact	33.076	0.500	32.274–36.256	159.547

In this diagnostic, the CPU-array/GPU-exact runtime ratio was 159.55 and the hybrid/GPU-exact ratio was 243.31. The separate 12.963 ms component is omitted from statistical-stage decomposition because its measurement definition could not be verified as a single test-plus-correction call. This limitation does not alter the independently timed complete backend call. Missing hybrid software versions are reported as not recorded. Source records: hybrid/summary.tsv and hybrid/metadata.json.

S5. Matched file workflows and batching

The matched file benchmark uses a deterministic synthetic background of 20,000 identifiers, a selected list of 500 identifiers and sets containing 32 genes. All six library sizes are retained. The boundary is input parsing through normalized file output; implementations use a one-sided greater test, the same filtered term family and BH correction, and the same five-field output contract. One warm-up precedes five timed repetitions; GPU calls are synchronized. OMP, OpenBLAS, MKL and NumExpr thread settings are 1. GOATOOLS is evaluated through its flat-GMT FisherFactory adapter, not an ontology-

propagation workflow. These inputs differ from the 35,158-gene real aggregate library used for the eight-stage profile.

Table S9 gives median matched-workflow times; the full IQRs, ranges and repetitions are supplied in matched/.

Table S9. Matched file workflow medians in milliseconds.

Gene sets	paraORA CPU	paraORA GPU	GSEApy CPU	GOATOOLS CPU
1,000	53.465	25.026	59.023	254.399
5,000	238.276	79.876	293.097	1270.390
10,000	499.986	186.469	592.533	2548.503
50,000	2528.619	967.959	3101.949	12710.861
100,000	5242.301	2053.073	6424.238	25528.275
141,002	7432.067	2917.803	9140.695	35965.525

Main Figure 5 shows IQRs from these repetitions and independently shows the real-library stages. Stage medians must not be used to decompose the synthetic file-to-file time, and their sum need not equal the median total time.

Table S10 summarizes a separate RTX 4090 batch experiment on the real 141002-set library, with a 35158-gene background and 500 genes per query. It measures prepared-query execution and does not include one-time library preparation or file writing.

Table S10. Best observed configuration in the batch benchmark.

Queries	Serial ms	Best batch	Best ms	Time reduction
1	80.106	1	76.350	4.69%
5	451.207	32	428.955	4.93%
10	939.738	4	913.462	2.80%
25	2223.003	4	2159.956	2.84%
50	4474.030	1	4323.150	3.37%
100	8940.316	16	8651.092	3.24%

The six candidate batch sizes were 1, 4, 8, 16, 32 and 100. The fastest observed median was selected separately at each query count; this post hoc selection includes batch size 1 and is not an unbiased estimate of a production parallel API benefit. Relative time reduction is 100 × (serial − best)/serial, ranging from 2.80% to 4.93%. Each configuration has five timing repeats. The archived numerical validation covers all 36 batch-size/query-count configurations. The production API processes queries serially. Serial timings belong to this batch experiment and are not pooled with the main repeated-query run.

Only overlap counting is batched: a single CUDA kernel evaluates the query-by-gene-set overlap matrix for each batch. Exact hypergeometric testing, BH adjustment, odds-ratio calculation, device-to-host transfer and result-frame assembly still run separately for each query. The limited reduction is therefore consistent with batching only one part of the prepared-query workflow; it does not establish a compute-bound or memory-bound bottleneck, or an upper limit on the benefit of a fully batched implementation. The timed calls also include sorting each result frame by term identifier and computing deterministic signature hashes for validation. The serial path explicitly synchronizes before and after each query; both paths are synchronized at the outer timing boundary. Library preparation,

kernel warm-up and file writing are excluded. These implementation and validation costs delimit the reported 2.80–4.93% reduction.

Sources: matched/timing_summary.tsv, matched/timing_repetitions.tsv, matched/agreement.tsv, batch/summary.tsv and batch/agreement_summary_validation.json.

S6. Recorded computing environments

Table S11 summarizes available metadata by experiment. Full JSON records retain platform strings, hardware inventories and additional settings. RTX 4090 provides the primary performance evidence. RTX 4060 Laptop and A40 extend the numerical evaluation and have archived eight-stage profiles; A40 also has a repeated-query timing series. Missing fields mean not recorded, not that a package or device was absent. Hybrid metadata are incomplete and are explicitly separated.

Table S11. Recorded software versions by experiment.

Experiment	Python	NumPy	SciPy	CuPy
425 cases 4090	3.12.3	2.5.1	1.18.0	14.1.1
425 cases 4060	3.12.0	2.5.2	1.18.1	14.2.0
425 cases A40	3.12.3	2.5.2	1.18.1	14.2.0
Repeated query 4090	3.12.3	2.5.1	1.18.0	14.1.1
Eight stage 4090	3.12.3	2.5.1	1.18.0	14.1.1
Matched files 4090	3.12.3	2.5.1	1.18.0	14.1.1
Batch 4090	3.12.3	2.2.4	1.18.0	14.1.1
Top500 4090	3.12.3	2.5.1	1.18.0	Not recorded
Hybrid diagnostic	3.12.3	2.2.4	Not recorded	Not recorded

The matched RTX 4090 environment records an Intel Core i9-13900K, 134,812,454,912 bytes of system RAM, CUDA runtime 12.9 and driver 595.84. It exposes one GPU for execution despite a two-device inventory. These fields are not imputed to experiments without their own records. The batch environment separately records NumPy 2.2.4. Device-level VRAM sampling in the archived capacity check reached 2,405 MiB; this is a sampled device-memory observation, not an allocator-level instantaneous peak.

Environment records are supplied in numerical/, repeated_query/, eight_stage/, matched/, batch/ and top500/. Hybrid metadata are supplied separately in hybrid/metadata.json. The limma analysis used R 4.5.2 and limma 3.66.0 on Windows; its session record is limma_R_sessionInfo.txt. These versions are not substituted for those of the GPU benchmarks. The same-limma external validation used clusterProfiler 4.18.4 in R 4.5.2; its full session record is validation_addendum/sessionInfo.txt. This version is distinct from clusterProfiler 4.20.0 in the earlier top-500 comparison. The A40 timing host used an Intel Xeon Gold 6148 CPU and Linux; the RTX 4060 Laptop timing host used Windows. Neither archive explicitly sets the OpenMP, MKL, OpenBLAS or NumExpr thread variables, so the RTX 4090 one-thread setting is not assigned to these runs. Experiment-specific timing environments are retained in validation_addendum/existing_gpu_timing/.

For reproducible single-thread CPU reference baselines, set OMP_NUM_THREADS, MKL_NUM_THREADS, OPENBLAS_NUM_THREADS and NUMEXPR_NUM_THREADS to 1 before starting Python, and record the package versions and hardware used. When these variables are unset, thread behavior depends on the numerical libraries and execution environment; unset values do not imply single-thread operation. This recommendation does not retrospectively change the archived A40 or RTX 4060 settings.

S7. Exploratory limma analysis

We reanalyzed the archived GSE19429 processed matrix [27] distributed with rapidGSEA [16]. The matrix contains 20,639 gene identifiers from 183 MDS samples and 17 controls. The archived conversion maps each probe to the first listed gene symbol and retains the per-sample maximum for probes assigned to the same gene. All intensities were positive; we applied log2 transformation without a pseudocount and did not reprocess the original CEL files.

Differential expression was assessed with limma 3.66.0 in R 4.5.2 [28] using a two-group model (MDS minus control), empirical-Bayes moderated t-tests with trend = TRUE, and no clinical or technical covariates. BH adjustment covered all 20,639 genes. Genes with adjusted $p \leq 0.05$ and an absolute mean log2-expression difference ≥ 1 were selected, yielding 13 upregulated and 69 downregulated genes. Direction-specific ORA used the 20,639 tested identifiers as background and the 50 Hallmark sets from MSigDB v5.0, with effective set-size limits of 1–5000 genes; correction was performed across all 50 terms within each direction. paraORA CPU probabilities and BH values were cross-checked with R phyper and p.adjust using the same contingency counts.

No Hallmark term was significant for the upregulated list. Six terms were significant for the downregulated list. The analysis is an exploratory software-use case; enrichment indicates over-representation of selected genes and does not establish pathway activity, a replicated MDS signature, or a clinical mechanism.

Table S12 lists all significant downregulated-list terms. Both directions were tested across all 50 terms; no term was excluded from BH correction because of a zero overlap.

Table S12. Significant Hallmark terms for the downregulated limma list.

Hallmark term	Overlap	Effective set size	p-value	BH-adjusted p
TNFα signaling via NF-κB	5	200	5.5250e-04	2.7625e-02
IL6–JAK–STAT3 signaling	3	87	3.1014e-03	3.7728e-02
IL2–STAT5 signaling	4	198	4.3693e-03	3.7728e-02
Hypoxia	4	200	4.5273e-03	3.7728e-02
Inflammatory response	4	200	4.5273e-03	3.7728e-02
Interferon-γ response	4	200	4.5273e-03	3.7728e-02

TNFα signaling via NF-κB: IL7R, NR4A2, NR4A3, PDE4B, PTPRE.

IL6–JAK–STAT3 signaling: CD9, DNTT, LTB.

IL2–STAT5 signaling: CD79B, IRF4, LRIG1, LTB.

Hypoxia: AKAP12, CTGF, ISG20, P4HA2.

Inflammatory response: IL7R, PDE4B, PTPRE, RGS1.

Interferon-γ response: IRF4, ISG20, P2RY14, PDE4B.

The maximum absolute differences from R phyper and p.adjust were 5.00e-16 for p-values and 2.33e-15 for BH-adjusted values, using the same contingency counts. This validates the probability and correction calculations, not independent gene mapping. The same lists were subsequently evaluated with clusterProfiler under aligned query-size and multiple-testing definitions, as described in Section S8. No GPU run on these limma-derived lists is claimed.

The full 20639-gene table is limma_gene_table.tsv. The 82 selected genes, their directions, effects and p-values are in limma_selected_genes.tsv. Direction-specific lists and both complete 50-term ORA tables are supplied alongside them.

S8. Independent comparison with clusterProfiler

A separate fixed 500-gene query was ranked by mean expression difference in the original source units. It was not selected by the limma procedure. paraORA CPU, paraORA GPU-exact and clusterProfiler 4.20.0 [13] used the same 20639-gene background and Hallmark mapping. clusterProfiler enricher used a forced universe, set-size limits of 1–5000, cutoffs of 1 and its full result slot. Comparisons were aligned by term identifier.

All three archived outputs contain the same 50 terms, overlap counts and effective set sizes, with 29 terms passing BH-adjusted $p \le 0.05$. Table S13 compares each paraORA output directly with clusterProfiler; FDR decisions also agree at 0.01 and 0.10.

Table S13. Agreement with clusterProfiler on the independent fixed top-500 query.

Candidate	Terms	Significant terms	Maximum $|\Delta p|$	Maximum $|\Delta BH|$
paraORA CPU	50	29	9.437e-16	1.110e-15
paraORA GPU-exact	50	29	1.983e-11	2.242e-11

Significance is evaluated at BH-adjusted $p \le 0.05$. Source outputs and the term-aligned comparison are supplied in top500/. The archived agreement.tsv uses paraORA CPU as its reference; comparison_to_clusterprofiler.tsv explicitly uses clusterProfiler as the reference for this table.

The same-limma external validation is provided in the Supplementary Validation Addendum (validation_addendum/). We ran clusterProfiler 4.18.4 on the archived 13-gene upregulated and 69-gene downregulated lists, the same 20,639-gene background and the same 50 MSigDB v5.0 Hallmark sets. The full background was forced, set-size limits were 1–5,000 and output cutoffs were 1. This version of enricher counts only query genes mapped to its annotations, reducing the effective query sizes to 6 and 29; it returns only positive-overlap terms and adjusts over those returned terms. With these native query and BH conventions and the full background forced, five upregulated-list terms and twelve downregulated-list terms were significant. These results define a different analysis from the full-query, 50-term paraORA analysis.

To match the original analysis, we added an auxiliary mapping set containing all background genes and excluded it by the 5,000-gene upper size limit. This retained the complete query sizes of 13 and 69 without altering any Hallmark membership or including the auxiliary set in the tested results. We restored zero-overlap Hallmark terms with $p = 1$ and applied R p.adjust over all 50 terms. This BH alignment was performed outside the native clusterProfiler result slot; the aligned values are not described as unmodified native output.

Under these aligned definitions, all 50 overlap counts and decisions at BH-adjusted $p \le 0.05$ agreed with paraORA CPU in each direction, retaining zero significant upregulated-list terms and the same six downregulated-list terms reported in Table S12 and main Figure 3. Maximum p/BH differences were 2.91×10^{-16} / 2.33×10^{-15} for the upregulated list and 5.00×10^{-16} / 8.88×10^{-16} for the downregulated list. The native outputs, full-query raw outputs and complete 50-term comparison tables are saved separately as *_clusterProfiler_native.tsv, *_clusterProfiler_full_query_raw.tsv and *_comparison_all50.tsv. validation_summary.tsv summarizes the results; run_validation.R, inputs/ and sessionInfo.txt permit reproduction.

S9. Repeated-query workflow timing

The repeated-query experiment used RTX 4090, 141002 real aggregate gene sets, a 35158-gene background and 100 deterministic distinct 500-gene lists. Checkpoints were 1, 5, 10, 25, 50 and 100 serial queries, with one warm-up and five measured repetitions. Table S14 reports the complete in-memory workflow, including one-time parsing and library preparation. Per-query execution includes

normalization, overlap counting, exact testing, BH correction, odds ratios, device-to-host transfer and result-frame construction; file writing is excluded.

Table S14. Complete in-memory runtime across repeated queries on RTX 4090.

Queries	CPU median (ms)	CPU IQR (ms)	GPU median (ms)	GPU IQR (ms)	CPU/GPU ratio
1	11417.968	68.412	6037.009	81.187	1.89
5	32773.706	101.307	6153.132	75.307	5.33
10	59178.987	128.855	6312.518	75.454	9.37
25	137005.999	144.553	6703.639	79.364	20.44
50	267156.274	346.095	7391.158	80.100	36.15
100	525791.662	306.326	8749.315	72.279	60.10

IQR is the 75th minus 25th percentile. The runtime ratio divides CPU median by GPU median. Prepared-query timing is separately preserved in repeated_query/checkpoint_summary.tsv; setup and checkpoint repetitions are supplied in the same folder. Sharing preparation costs reduces average workflow time per query; it does not reduce the amount of statistical work required for each query. The archived A40 repeated-query experiment used the same library size, background size, query-size and checkpoint protocol, with one warm-up and five timed repetitions. Median CPU/GPU complete in-memory times were 31.267/13.619 s for one query and 1,685.703/19.426 s for 100 queries, yielding within-host ratios of 2.30 and 86.78. Preparation was included and file writing excluded. All checkpoint medians were reproduced from the archived repetitions. Host and software differences prevent interpreting these ratios and the RTX 4090 ratios as a ranking of GPUs. The full checkpoint summary, repetitions, environments and sentinel records are in validation_addendum/existing_gpu_timing/R1_Full_Query_Scaling_A40_20260902/.

S10. Eight-stage workflow profile

Table S15 reports an independent RTX 4090 profile with the same real library and background sizes and one 500-gene query. The profile used one complete warm-up and five timed repetitions, with GPU synchronization before and after each GPU stage. Process startup, imports, environment creation and initial CUDA-context creation were excluded. Each stage was measured separately.

Table S15. Eight-stage timing for the real aggregate library on RTX 4090.

Stage	Median (ms)	IQR (ms)	Minimum (ms)	Maximum (ms)	Share (%)
GMT parsing	1146.222	22.719	1130.244	1269.146	16.411
Library preparation	4932.303	4.535	4886.264	4958.156	70.618
Host-to-device transfer	6.215	0.016	6.205	6.645	0.089
Overlap counting	2.257	0.008	2.250	2.277	0.032
Hypergeometric testing	4.591	0.026	4.555	4.681	0.066
BH correction	0.788	0.006	0.775	0.815	0.011
Result assembly	15.194	1.053	14.503	16.077	0.218
File writing	876.871	32.957	526.487	1030.195	12.555

Result assembly includes odds-ratio calculation, device-to-host transfers and DataFrame construction in this profile. Shares divide each stage median by the sum of the eight stage medians; that sum is not the median total workflow time. This real-library profile is distinct from the synthetic matched-tool workload, which has 20000 background identifiers and 32 genes per set. Source: eight_stage/stage_summary.tsv. Archived RTX 4060 Laptop and A40 profiles used the same eight stages, library, background and 500-gene query, with one warm-up and five timed repetitions. Library preparation accounted for 76.8% and 74.8%, respectively, of the sum of stage medians. The RTX 4060 overlap stage ranged from 15.413 to 262.707 ms, so dispersion must be considered alongside its median. Original medians were reproduced from the raw repetitions. Both profiles retained raw-p tolerance failures with a maximum absolute difference of approximately 8.72×10^{-11}; overlaps, BH values and decisions at 0.01, 0.05 and 0.10 agreed. Full summaries, repetitions,

agreement records and environments are in validation_addendum/existing_gpu_timing/R1_Stage_Profile_RTX4060_20260902/ and R1_Stage_Profile_A40_20260902/. These cross-host timings are descriptive and do not isolate GPU effects.

Supplementary Note S1. Local use and reporting conventions

With paraORA installed, run the following command from the accompanying Supplementary_Data directory. All three input files are included. The CPU example requires no GPU; it uses the exact hypergeometric test and retains the full set of 50 terms.

```
paraora ora --de-genes limma_downregulated_genes.txt --background background.txt --gmt
h.all.v5.0.symbols.gmt --method hypergeom --backend cpu --min-size 1 --max-size 5000 --output
example_cpu.tsv
```

To request GPU-exact execution in a compatible CUDA/CuPy environment, replace --backend cpu with --backend gpu-exact and use a different output filename. An explicit GPU request fails when CUDA is unavailable; auto may select CPU. The optional GPU command has not been executed for these limma lists. Use cpu for reference execution or cpu-vectorized for CPU array evaluation. The gpu-exact path keeps the principal statistical stages on the device, whereas gpu-hybrid is primarily diagnostic and evaluates statistics on the host. The auto mode selects GPU-exact when CUDA is available and otherwise uses CPU. Inspect the available execution metadata described below to confirm the path used.

Local ORA on the three supplied input files does not require an external Web API once paraORA and its dependencies are installed. Installation and acquisition of other third-party datasets or gene-set collections may require network access and must follow their access and licensing conditions. Reproducing the larger performance experiments additionally requires their documented input resources and compatible hardware. Repository documentation at docs/reproducibility.md maps experiments to scripts and inputs; docs/usage.md provides installation and usage instructions. The authors commit to maintaining paraORA for at least five years from the date of publication. The maintenance policy is recorded in MAINTENANCE.md in the repository.

The following three records are abbreviated displays of archived downregulated-list results, not newly executed CLI output. Values are rounded here; all fields and full precision remain in limma_down_paraora_all50.tsv.

```
HALLMARK_TNFA_SIGNALING_VIA_NFKB
overlap=5; gene_set_size=200; p_value=5.525015e-04; fdr=2.762507e-02
```

```
HALLMARK_IL6_JAK_STAT3_SIGNALING
overlap=3; gene_set_size=87; p_value=3.101395e-03; fdr=3.772765e-02
```

```
HALLMARK_IL2_STAT5_SIGNALING
overlap=4; gene_set_size=198; p_value=4.369274e-03; fdr=3.772765e-02
```

The archived tables use term, description, overlap, gene_set_size, p_value, fdr, odds_ratio and hit_genes. Their execution context is documented separately in the case records; no backend column has been added to those files. The current CLI uses term_id and set_size and writes additional fields, including n_background, n_de, backend_used and actual_backend. The scalar CPU path identifies itself through backend_used = cpu_reference; actual_backend can remain unspecified on that path. Retain both the explicit command and available execution fields when recording an analysis.

Identifier matching is exact and case-sensitive. Leading/trailing whitespace is removed and duplicates are removed stably, but aliases, identifier namespaces, and species are not inferred or converted. Users must harmonize identifiers before analysis. Queries and gene sets are intersected with the explicit background; outside-background query identifiers are reported and excluded. Empty post-filter

queries, libraries with no retained sets, and invalid contingency tables produce errors rather than results. A retained term with zero overlap is a valid test with $p = 1$. Odds ratios use $a = k$, $b = n - k$, $c = S - k$ and $d = N - n - S + k$. The implementation returns infinity when $bc = 0$ and $a > 0$, 1 when $bc = 0$ and $a = 0$, and 0 when $bc > 0$, $a = 0$, and $d > 0$; when $bc > 0$, $a = 0$ and $d = 0$, it returns 1. In all other valid cases it returns $ad/(bc)$. These are reporting conventions, not measured null effects.

S11. Web-service observations

The public HTTPS deployment audited on 1 September 2026 has no application-level authentication or rate limiter. Inspected application logging covers standard request metadata rather than submitted gene contents. GMT/background uploads use per-session temporary directories with a nominal six-hour inactivity TTL, but cleanup is triggered by later session access rather than guaranteed at a fixed deadline. Completed job inputs and results remain in process memory until restart. Upload maxima are 256 MiB (GMT), 32 MiB (background), and 8 MiB (gene list); the deployed process runs at most two jobs concurrently and paginates result retrieval to 1,000 rows per request while permitting full TSV/CSV download. A single public smoke-test query returned ten terms with a reported analysis elapsed time of 2.854 s; this is a functional probe, not a latency guarantee. Do not submit confidential or identifiable data; use local execution for sensitive work.

S12. Supplementary data and source records

Supplementary_Data contains the full gene-level limma results, selected genes, both 50-term ORA tables, the independent top-500 comparisons and the numerical and performance records cited above. File paths in this document are relative to that directory. README.md explains the fields and workload boundaries; manifest.tsv lists file checksums and provenance. Absolute paths retained inside historical metadata describe the original runs and are not required to use the supplied inputs. The integrated Supplementary Validation Addendum resides in validation_addendum/. It contains both new CPU-side external validation and copies of previously executed GPU timing records; no new GPU or limma-model run was performed. The original 60 manifest-listed data files are unchanged. README.md and manifest.tsv are updated to index the complete package, and their previous versions are preserved under provenance/. Historical statements such as external_validation pending in archived metadata describe the earlier evidence state; validation_addendum/validation_summary.tsv and the present supplement document the subsequent validation. Source checksum lists inside copied historical subsets may refer to files retained only in the full source archives; the top-level manifest.tsv covers the actual delivered files.

The reference numbers used here are shared with the main manuscript: rapidGSEA [16], GSE19429 [27], limma [28] and clusterProfiler [13].
